# Pre-hypertension in Uganda: a cross-sectional study

**DOI:** 10.1186/1471-2261-13-101

**Published:** 2013-11-14

**Authors:** Fred Nuwaha, Geofrey Musinguzi

**Affiliations:** 1 Department of Disease Control and Environmental Health, School of Public Health, College of Health Sciences, Makerere University, P.O. Box 7072, Kampala, Uganda

**Keywords:** Cardiovascular diseases, Non communicable diseases, Low income countries, Risk factors, Prevalence

## Abstract

**Background:**

Persons with a systolic blood pressure (BP) of 120 to < 140 or diastolic BP of 80 to < 90 mm hg are classified as having pre-hypertension. Pre-hypertension is associated with cardiovascular disease (CVD) risk factors, incident CVD and CVD mortality. Understanding determinants of pre-hypertension especially in low income countries is a pre-requisite for improved prevention and control.

**Methods:**

Data were analyzed for 4142 persons aged 18 years and older with BP measured in a community cross sectional survey in Uganda. The prevalence of pre-hypertension was estimated and a number of risk factors e.g. smoking, use of alcohol, overweight, obesity, physical activity, sex, age, marital status, place of residence, and consumption of vegetables and fruits were compared among different groups (normotension, pre-hypertension, and hypertension) using bivariate and multivariable logistic regression.

**Results:**

The age standardized prevalence of normal blood pressure was 37.6%, pre-hypertension 33.9%, hypertension 28.5% and raised blood pressure 62%. There was no difference between the prevalence of hypertension among women compared to men (28.9% versus 27.9%). However, the prevalence of pre-hypertension was higher among men (41.6%) compared to women (29.4%). Compared to people with normal blood pressure, the risk of pre-hypertension was increased by being 40 years and above, smoking, consumption of alcohol, not being married, being male and being overweight or obese. Compared to pre-hypertension, hypertension was more likely if one was more than 40 years, had infrequent or no physical activity, resided in an urban area, and was obese or overweight.

**Conclusions:**

More than one in three of adults in this population had pre-hypertension. Preventive and public health interventions that reduce the prevalence of raised blood pressure need to be implemented.

## Background

Pre-hypertension is defined as a systolic blood pressure (SBP) of 120 to less than 140 mmHg or diastolic blood pressure (DBP) of 80 to less than 90 mmHg in patients not on medication for hypertension [1]. Pre-hypertension is common in both low and high income countries and is associated with cardiovascular disease (CVD) risk factors, incident CVD and CVD mortality [2-10].

For people above 40 years the complications associated with increasing blood pressure begin at a SBP/DBP of 115/75 mm Hg, with the risk of cardiovascular disease doubling with each increment of 20/10 mm Hg [10]. Modifications of lifestyle such as losing weight, increased physical activity reduction in consumption of alcohol and modified diet for all people with pre-hypertension and the addition of drug therapy for patients who have other compelling indicators, including multiple diseases are recommended for control of pre-hypertension in high income countries [1]. The application of these guidelines is difficult to implement in low income countries such as Uganda and much of sub-Saharan Africa where detection, awareness, treatment and control of hypertension is less than optimal [11]. Awareness of hypertension largely depends on the capacity of the health system to provide diagnostic services for hypertension to the general population [11,12]. Unfortunately, the healthcare system in Uganda is largely constrained by communicable diseases and non communicable diseases (NCDs) including pre-hypertension/hypertension are still largely not routinely detected [13].

Studies estimating frequency of pre-hypertension in Uganda and Sub Saharan Africa are scarce, despite the reported increase in the prevalence of hypertension in the region [14,15]. Information on frequency of pre-hypertension and other NCDs is needed for planning, prioritization and to provide evidence to use in mobilization of resources [16,17]. This study estimated the prevalence of pre-hypertension and associated risk factors in Buikwe and Mukono districts of Uganda. The predictors reported include socio-demographic factors that identify segments of population at increased risk of hypertension/pre-hypertension and those that can be modified by life style interventions.

## Methods

### Setting

The data used for this analysis was derived from a cross-sectional survey conducted in Mukono and Buikwe districts of Uganda using a complex, stratified, multistage probability cluster sampling design whose details have been reported elsewhere [11]. In this analysis, 4142 adult men and women 18 years or older with complete data on BP measures were included. Blood pressure (BP) was measured at participant’s homes on a single occasion using automated digital blood pressure monitor, model LD7 with appropriate cuff sizes. Three blood pressure measurements (at least 1 minute apart) were with the participant seated, using a calibrated automated digital BP machine. Before the first reading a five minutes rest was allowed. For each participant, the mean of the three values was calculated to estimate their blood pressure. BP was measured on all participants during a home examination by trained research assistants.

### Study variables

#### Blood pressure levels

Participants were separated into the following 4 groups based on their measured BP (average SBP and DBP, (1) normal blood pressure: SBP less than 120 mm Hg and DBP less than 80 mm Hg; (2) pre-hypertension (not hypertension): SBP of 120 mm Hg or higher but lower than 140 mm Hg or DBP of 80 mm Hg or higher but lower than 90 mm Hg; (3) hypertension: SBP of 140 mm Hg or higher or DBP of 90 mm Hg or higher or taking antihypertensive medication; and (4) increased BP: all those who had pre-hypertension or hypertension.

#### Body weight status

Based on measured weight and height data collected, body mass index (BMI; a measure of weight in kilograms divided by the square of height in meters) was calculated for each participant. Participants were grouped into 2 categories: (1) nonoverweight (BMI less than 25); (2) overweight/obese (BMI of 25 or more but less than 30 for overweight and obese BMI of 30 or higher). Women who reported that they were pregnant were excluded in the analysis stratified by body weight status.

#### Age

For most age-related comparisons, participants were separated into 2 groups (below 40 and ≥ 40 years). It was not possible to use more groups due to the relatively small sample size of the survey. Furthermore because of the low life expectancy in Uganda, it was not possible to have adequate numbers of people above 60 years as only 10.2% of the sample was above 60 years.

#### Education status

People reported education levels were classified as never attended school, attended primary, attended secondary and more than secondary. This was later collapsed into two categories of none/primary and secondary or above to assess the influence of education on blood pressure.

#### Marital status

Marital status was classified as never married, currently married or cohabiting, divorced, separated or widowed. For analysis two categories of currently married and others were used.

#### Smoking status and use of alcohol

Participants were asked if they currently consume tobacco and or its products and whether they currently consume alcoholic beverages.

#### Consumption of vegetables and fruits

Consumption of fruits and vegetables was measured by asking people how many times they consumed vegetables and or fruits in the previous week. Those who reported at least 7 times were classified as frequent consumers whereas those who consumed less than 7 times were classified as infrequent consumers.

#### Residence

People were classified as urban dwellers if they resided in a town with a population of more than 10,000 persons as defined by the Uganda national bureau of statistics [18]. Others who did not meet this criterion were classified as rural dwellers.

#### Physical activity

Participants were asked how many times they got involved in physical activity such as walking, riding, manual work, exercises and sports in a week. Those who reported at least 3 times were classified as frequent whereas those who reported less than three times a week were regarded as infrequent.

### Statistical analysis

All analyses took into account the complex cluster sample design. Estimate of the standard errors were calculated with a robust standard errors and cluster option in the STATA 10.0 software (Texas, USA). The prevalence of normal BP, pre-hypertension, hypertension and raised BP was age standardized using World Health Organization (WHO) world population. To guage whether increasing blood pressure differed with age, level of education, BMI, consumption of vegetables and fruits and whether distribution of blood pressure differed by marital status, smoking, use of alcohol, and place of residence, a Cochran-Armitage chi-square for linear trend was used to test associations among groups of normal BP, pre-hypertension and hypertension. To identify independent predictors of pre-hypertension two analyses were made. First people with normal BP were compared with those with pre-hypertension. Second people with pre-hypertension were compared with those with hypertension. The crude odds ratios (COR) were compared to adjusted odds ratios (AOR) and their 95% confidence intervals (CI) after multivariable logistic regression analysis. In the multivariable logistic regression analysis predictor variables were controlled for age, sex, marital status, education status, consumption of alcohol, place of residence as well as consumption of fruits and vegetables. The prevalence of normal BP, pre-hypertension, hypertension and abnormal BP was assessed by calculating proportions with their CI. Significance for proportions was tested using a two tailed Pearson’s chi-square test with Yates’s collection. For all tests, a p-value of < 0.05 was taken as statistically significant.

### Ethics statement

The study was approved by Makerere University School of Public Health institutional review board and the Uganda National Council of Science of Technology. Written informed consent was obtained from the participants. People diagnosed with hypertension were referred to health units.

## Results

Of the 4142 study participants that were analyzed, 1477 (35.7%) were men. The overall mean age was 36.5 (15.2) years. Men with a mean age of 38.1 and a standard deviation 16.2 years were older than women with a mean age 35.6 (14.5) years. The other main characteristics of the survey participants are provided in Table 1. About two thirds of the study participants were below 40 years with more women than men (66% versus 59%) being less than 40 years. Approximately one third were urban dwellers and place of residence was the same among women and men. Men were better educated compared to women with 48% of men having attained secondary or higher compared to 40% of the women. More than 60% of the people were either married or cohabiting and the proportion of married people was similar among men and women. About one in twenty of the population was current smokers and more men than women smoked. Thirty seven percent of men and 20% of women reported to be current consumers of alcohol. Twenty percent of the study participants were either obese (6%) or over weight (14%), 84% were frequently physically active, 54.2% frequently consumed vegetables, and 40.9% frequently consumed fruits. More women than men were overweight (18% versus 7%) or obese (8.5% versus 1.5%). On the other hand, more men than women frequently consumed vegetables (48.7% versus 44.3%) and were more active (86.2% versus 82.3%). There was no difference between women regarding consumption of fruits (41.4% versus 39.9%).

**Table 1:** Distribution of studied variables by sex among survey participants

**Variable**	**Total**	**Female**	**Male**	**P-value**
Age in years				
Below 40	2628 (63.4)	1762 (66.1)	866 (58.6)	
40-59	1090 (26.3)	666 (25.0)	424 (28.7)	
Above 60	424 (10.2)	237 (8.9)	187 (12.7)	< 0.001
Place of residence				
Urban	1369 (33.1)	893 (33.5)	476 (32.2)	
Rural	2773 (66.9)	1772 (66.5)	1001 (67.8)	0.42
Education level				
None/primary	2375 (57.3)	1609 (60.4)	766 (51.9)	
Secondary/tertiary	1767 (42.7)	1056 (39.6)	711 (48.1)	< 0.001
Marital status				
Currently married	2573 (62.1)	1668 (62.6)	905 (61.3)	
Not married	1767 (42.7)	997 (37.4)	572 (38.7)	0.40
Currently smokes				
Yes	267 (6.4)	52 (2.0)	215 (14.6)	
No	3875 (93.6)	2613 (98.0)	1262 (85.4)	< 0.001
Currently consumes alcohol				
Yes	1068 (25.8)	528 (19.8)	540 (36.6)	
No	3074 (74.2)	2137 (80.2)	937 (63.4)	< 0.001
Body mass index (Kg/M^2^)				
Less than 25	3305 (79.8)	1952 (73.2)	1353 (91.6)	
25 to less than 30	589 (14.2)	487 (18.3)	102 (6.9)	
30 and above	248 (6.0)	226 (8.5)	22 (1.5)	< 0.001
Physical activity				
Frequent	3465 (83.7)	2192 (82.3)	1273 (86.2)	
Infrequent/none	677 (16.3)	473 (17.7)	204 (13.8)	0.001
Fruit consumption				
Infrequent	2449 (59.1)	1562 (58.6)	887 (60.4)	
Frequent	1693 (40.9)	1103 (41.4)	590 (39.9)	0.37
Vegetables consumption				
Infrequent	1899 (45.8)	1180 (44.3)	719 (48.7)	
Frequent	2243 (54.2)	1485 (55.7)	758 (51.3)	0.007

P-value relates to differences between men and women.

Numbers in parentheses are percentages.

### Prevalence of abnormal blood pressure

The age standardized prevalence of normal blood pressure was 37.6%, pre-hypertension 33.9%, hypertension 28.5% and raised blood pressure 62% (Table 2). There was no difference between the prevalence of hypertension among women compared to men (28.9% versus 27.9%). However, the prevalence of pre-hypertension was higher among men (41.6%) compared to women (29.4%). As a consequence, more women than men had normal blood pressure (41.7% versus 30.5%) whereas more men than women had raised blood pressure (67.5% versus 58.3%). The prevalence of non-standardized rates follows a similar trend as for standardized rates (Table 2).

**Table 2:** Distribution of normal blood pressure pre-hypertension and hypertension by sex

**Variable**	**Total (N = 4142) % (CI)**	**Women (N = 2665) % (CI)**	**Men (N = 1477) % (CI)**	**P-value**
Normal BP	41.5 (40.0-43.0)	46.8 (44.8-48.7)	32.1 (29.7-34.5)	< 0.001
Pre-hypertension	35.2 (33.8-36.7)	30.5 (28.9-32.3)	43.7 (41.2-46.3)	< 0.001
Hypertension	23.2 (21.9-24.5)	22.7 (21.1-23.4)	24.2 (22.0-26.4)	0.30
Raised BP*	58.5 (57.0-60.0)	53.2 (51.3-55.1)	67.9 (65.4-70.3)	< 0.001
Age standardized prevalence				
Normal BP	37.6 (36.1-39.1)	41.7 (39.8-43.6)	30.5 (28.2-32.8)	< 0.001
Pre-hypertension	33.9 (32.0-34.9)	29.4 (27.7-31.2)	41.6 (39.2-44.1)	< 0.001
Hypertension	28.5 (27.2-29.9)	28.9 (27.2-30.6)	27.9 (25.6-30.2)	0.64
Raised BP*	62.0(60.5-63.4)	58.3 (56.3-60.2)	69.5 (67.2-71.8)	< 0.001

*Includes pre-hypertension and hypertension.

P-value is for the difference between women and men.

The distribution of blood pressure by studied variables is shown in Table 3. Blood pressure increased with increasing age, increased consumption of alcohol, increased level of smoking, increasing body mass index and increased residence in an urban area. On the other hand blood pressure decreased with increasing consumption of vegetables and fruits, increasing rate of marriage and increasing level of having attained secondary education.

**Table 3:** Distribution of normal blood pressure pre-hypertension and hypertension by age, education, marital status, diet, physical activity, smoking, use of alcohol, BMI and place of residence

**Variable**	**Total**	**Normol BP**	**Pre-hypertension**	**Hypertension**	**(Chi-square)**
	**(N = 4142) % (CI)**	**(N = 1720) % (CI)**	**(N = 1460) % (CI)**	**(N = 962) % (CI)**	**P-value**
Was 40 years and above					
	36.6 (35.1-38.1)	23.5 (21.5-25.5)	33.5 (31.1-35.9)	64.7*(61.7-66.7)	(413.7) < 0.001
Reported frequent physical activity					
	83.7 (82.6-84.8)	85.3 (83.6-87.0)	85.4 (83.6-87.2)	78.0 (75.4-80.6)	(9.2) < 0.001
Reported frequent consumption of vegetables					
	45.8 (44.3-47.3)	47.5 (45.1-49.9)	45.7 (43.1-48.3)	43 0 (40.0-46.1)	(4.7) 0.030
Reported frequent consumption of fruits					
	40.9 (39.4-42.4)	42.6 (40.3-44.9)	41.9 (39.4-44.3)	36.2 (33.2-39.3)	(9.2) 0.002
Currently consumes Alcohol					
	25.8 (23.4-28.1)	21.7 (19.0-24.4)	27.1 (24.2-29.8)	31.2 (27.2-35.2)	(30.8) < 0.001
Currently smokes					
	6.4 (5.4-7.5)	5.8 (4.6-6.9)	6.2 (4.6-7.9)	8.0 (6.2-9.8)	(4.7) 0.030
Was married or co-habiting					
	62.1(60.6-63.6)	66.5 (64.3-68.7)	59.9 (57.4-62.4)	57.6 (54.5-60.7)	(23.7) < 0.001
Had at least secondary level of education					
	42.7 (41.2-44.2)	45.3 (43.0-47.7)	44.9 (42.4-47.5)	34.5 (31.5-37.5)	(24.5) < 0.001
Body Mass Index is 25 Kg/M^2^ or more					
	20.2 (19.0-21.4)	15.5 (13.8-17.2)	19.7 (17.7-21.7)	29.3 (26.4-32.2)	(68.9) <0.001
Sex is male					
	35.7 (34.2-37.2)	27.6 (25.5-29.7)	44.2 (41.6-46.8)	37.1 (34.1-40.2)	(40.3) < 0.001
Resided in an urban area					
	33.1 (31.7-34.5)	31.0 (28.8-33.2)	33.5 (31.1-35.9)	36.0 (33.0-39.0)	(6.9) 0.008

P-value refers to test of chi-square for linear trend among those with normal blood pressure, pre-hypertension or hypertension.

*Numbers shown in columns are percent of people with normal BP, pre-hypertension or hypertension that have a particular variable. E.g. 64.7% of people with hypertension are 40 years and above.

### Independent predictors of pre-hypertension

Compared to people with normal blood pressure, the risk of pre-hypertension was increased by being 40 years and above, smoking, consumption of alcohol, not being married, being male and being overweight or obese. Compared to pre-hypertension, hypertension was more likely if one was more than 40 years, reported infrequent or no physical activity resided in an urban area and was obese or overweight. It is interesting that being male increases the chances of developing pre-hypertension whereas being female increases the chance of developing hypertension if one is already pre-hypertensive. However, the effect of sex on the pre-hypertension/hypertension comparison was only manifest at univariate but not at multivariable level (Table 4).

**Table 4:** Predictors of pre-hypertension compared to normal blood pressure and to hypertension

**Variable**	**Normal BP versus pre-hypertension**	**Pre-hypertension versus hypertension**		
	**COR (CI)**	**AOR (CI)**	**COR (CI)**	**AOR (CI)**
Age is 40 years and more				
	1.63 (1.39-1.90)***	1.51 (1.27-1.79)***	3.65 (3.08-4.34)***	3.47 (2.87-4.19)***
Reported infrequent or no physical activity				
	1.00 (0.82-1.23) ns	-	1.66 (1.34-2.04)***	1.31 (1.05-1.63)*
Reported infrequent consumption of fruits				
	1.02 (0.84-1.12) ns	-	1.27 (1.07-1.51)**	-
Reported infrequent consumption of vegetables				
	1.08 (0.94-1.24) ns	-	1.11 (0.94-1.31)	-
Reported current consumption of alcohol				
	1.34 (1.14-1.57)***	1.19 (1.01-1.41)*	1.32 (1.21-1.86)**	-
Reported current smoking				
	1.09 (0.80-1.47) ns	1.61 (1.18-2.18)**	1.11 (0.77-1.52) ns	-
Not currently married or co-habiting				
	1.32 (1.15-1.54)***	1.33 (1.12-1.58)***	1.10 (0.93-1.30) ns	-
Body Mass Index was 25 Kg/M^2^ or more				
	1.34 (1.11-1.62)**	1.69 (1.42-2.01)***	1.67 (1.40-2.04)***	1.52 (1.25-1.84)***
Had not attained secondary level of education				
	1.04 (0.86-1.13)	-	1.55 (1.30-1.84)**	-
Sex (male or female)				
	μ2.09 (1.79-2.43)***	μ 2.26 (1.90-2.71)***	∫1.34 (1.14-1.59)**	-
Resided in an urban area				
	1.12 (0.96-1.30) ns	-	1.12 (0.94-1.33) ns	1.38 (1.14-1.68)**

COR crude odds ratios; AOR adjusted odds ratios; CI confidence interval.

Ns P > 0.05; *P < 0.05; **P < 0.01; ***P < 0.001.

μ female is the reference sex; ∫ male is the reference sex.

## Discussion

This study shows that more than one in three of adults in this population have raised blood pressure in form of pre-hypertension. Pre-hypertension was more common among men compared to women. The other independent predictors of being pre-hypertensive compared to having normal blood pressure were being obese or overweight, being unmarried, smoking, consumption of alcohol and being over 40 years of age. The prevalence of pre-hypertension in our study is comparable with other studies elsewhere in both low and high income countries. Prevalence estimates reported range from 31% in the United States [3], 31.6% in Korea [4], 34% in Taiwan [6], 35% in Jamaica [9], 40% in the Ashanti region of Ghana [8], to 47% in Liaoning Province in China [7] and 48.9% among the military in Israel [5].

It was interesting to note that our results regarding the prevalence of increased blood pressure and of pre-hypertension were similar to what was reported from the United States in 2004 of data collected in 1999–2000 [3]. Although the rates of raised BP were similar, the risk factors e.g. aged population, increasing body mass index, urbanization, smoking, and consumption of alcohol were all much higher in USA compared to Uganda. It is possible that racial/ethnic factors may be responsible for these differences [19,20] as African-Americans in USA were more likely to have raised blood pressure due to genetic, dietary and factors related to salt sensitivity [21,22]. All study subjects in our sample were Africans.

Similar to what has been observed in other studies men were more likely to be pre-hypertensive compared to women [4-9]. This observation may be because women are protected by hormonal factors or factors related to pregnancy and child birth [23,24].

In our study people who were married or co-habiting were protected from pre-hypertension compared to never married, widowed separated or divorced and this may be related to lower levels of stress associated with married life. Marital differences in psychological status (prolonged stress and low social support), dietary intake (mainly sodium and potassium intake) and economic aspects of living alone are suggested as factors, which might explain at least partly the marital diversity in blood pressure and the risk of hypertension in men [25-27].

The finding of high levels of pre-hypertension in a low income country like Uganda present challenges for control and prevention. First it is unlikely that a low income country would afford pharmacological interventions for pre-hypertension due to prohibitive costs and the sheer magnitude of the problem. Even in high income countries these interventions are deemed not feasible [28]. Besides, treatment/control for hypertension and other non communicable diseases is still rudimentary in Uganda and other low income countries [11,13]. This means that it would be difficult to prioritize pre-hypertension when hypertension is not being adequately taken care of. Second lifestyle interventions are difficult to implement and maintain particularly among poor countries of the world [29-31]. Third low income countries like Uganda still have a huge burden of infections communicable diseases and the health systems and health financing are overwhelmed with control and treatment of these diseases. Thus emerging non communicable diseases will result in double jeopardy [11,13].

Because secondary prevention of pre-hypertension (through early diagnosis and treatment) is unfeasible in low income countries, primary preventions methods that limit the development of non communicable diseases and primordial prevention methods that reduce adoption of risk behaviors for non communicable diseases should be given priority [17,32,33]. As the epidemiological transition is in early stages in low countries these two prevention methods that are more likely to be cost-effective [34] should be .should be implemented without further delay. As measures to operationalise prevention of pre-hypertension are not widespread in low income countries, [11,13,28] operation research is urgently needed to define how best to implement the primary as well as the primordial prevention measures at community and national levels.

### Strengths and limitations

The limitations of our study include the cross-sectional nature meaning that causal inferences are difficult. There was a possible selection bias in the study due to the fact that most men were not found at home during the survey. In spite of these limitations, our sample size was big and we standardized the prevalence of estimates using a reference population.

## Conclusions

More than one in three of adults in this population have pre-hypertension. Cost effective primordial and primary prevention methods of reducing pre-hypertension should be implemented without further delay. Operations research to define the best ways of implementing prevention methods for pre-hypertension is urgently needed.

## Abbreviations

BP: Blood pressure; SBP: Systolic blood pressure; DBP: Diastolic blood pressure; CVD: Cardio vascular disease; NCD: Non communicable disease; CI: 95% confidence interval; COR: Crude odds ratio; AOR: Adjusted odds ratio; BMI: Body mass index.

## Competing interests

The authors declare that they have no competing interests.

## Authors’ contributions

FN conceived and designed the survey, performed statistical analyses and wrote the manuscript; and is the guarantor for the manuscript.GM conducted the survey assisted with statistical analyses, reviewed manuscript and interpretation of data. Both authors approved the final manuscript.

## Authors’ information

FN is an associate professor of diseases control and prevention at Makerere University, School of public health. His major research interests are optimization of measures for disease control in low income countries. GM is a research fellow at Makerere University School of Public Health. He has a background in environmental health and is interested in making sure that proven cost effective methods of disease control reach the people that need them.

## Pre-publication history

The pre-publication history for this paper can be accessed here:

http://www.biomedcentral.com/1471-2261/13/101/prepub
